# Supplementary material for: Altered microRNA Transcriptome in Cultured Human Liver Cells upon Infection with Ebola Virus
Source: Int J Mol Sci. 2021 Apr 6;22(7):3792. doi: 10.3390/ijms22073792 (PMC8038836; doi:10.3390/ijms22073792)
Supplement: Supplementary file 1 [file ijms-22-03792-s001.zip › Supplementary_File/C_ GO_Analysis_Results/16-30nt_go_Reston-24h-Huh7_vs_Control-24h-Huh7_up.mature_mirna_targets/CC_FoldEnrichmentDotPlot.pdf]

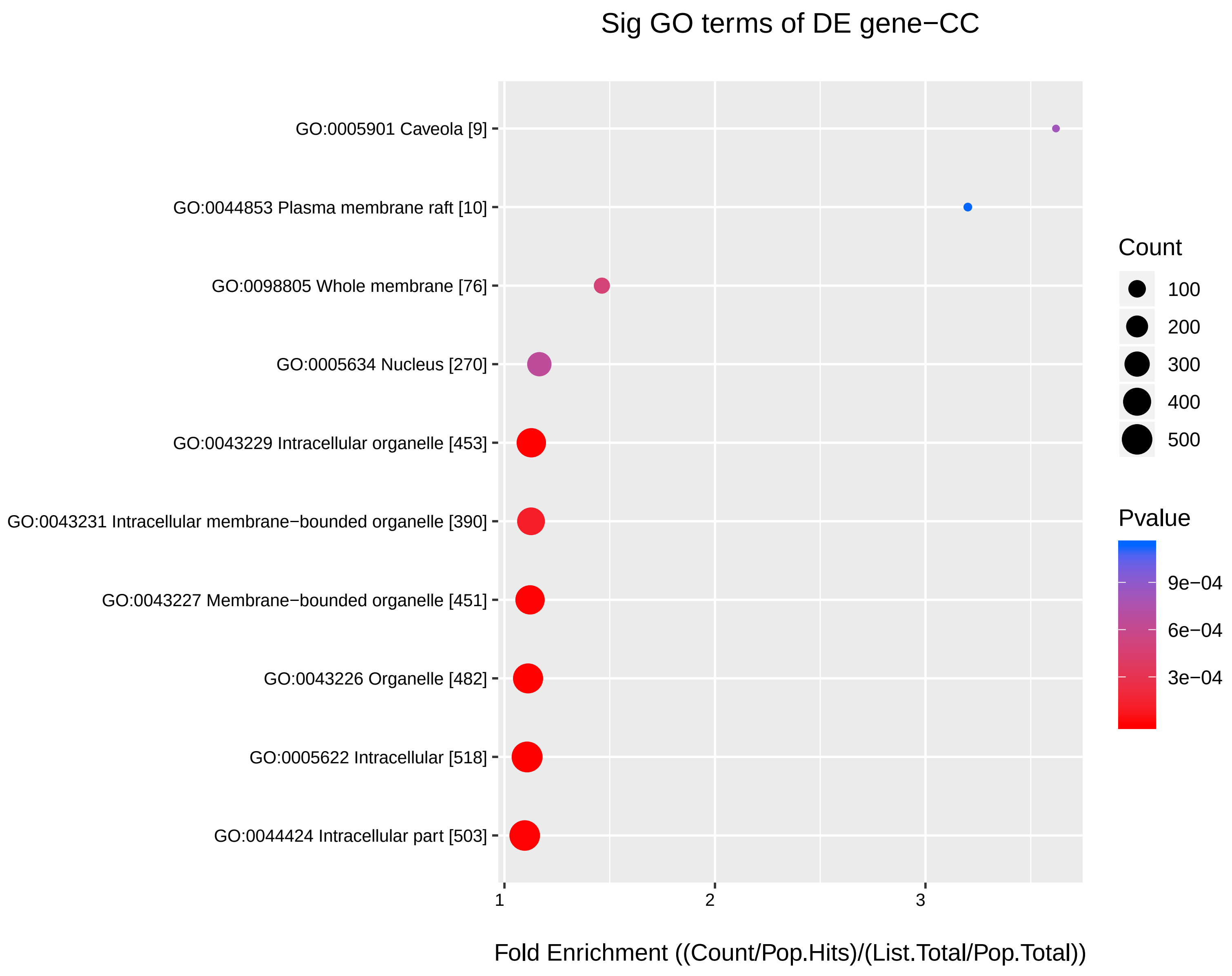
Sig GO terms of DE gene−CC
GO:0005901 Caveola [9]
GO:0044853 Plasma membrane raft [10]
GO:0098805 Whole membrane [76]
GO:0005634 Nucleus [270]
GO:0043229 Intracellular organelle [453]
GO:0043231 Intracellular membrane−bounded organelle [390]
GO:0043227 Membrane−bounded organelle [451]
GO:0043226 Organelle [482]
GO:0005622 Intracellular [518]
GO:0044424 Intracellular part [503]
1
2
3
Fold Enrichment ((Count/Pop.Hits)/(List.Total/Pop.Total))
Count
100
200
300
400
500
Pvalue
9e−04
6e−04
3e−04
